# Supplementary material for: The causal role of circulating inflammatory markers in osteoporosis: a bidirectional Mendelian randomized study
Source: Front Immunol. 2024 Jul 18;15:1412298. doi: 10.3389/fimmu.2024.1412298 (PMC11291241; doi:10.3389/fimmu.2024.1412298)
Supplement: Supplementary file 1 [file DataSheet_1.zip › supplementary material/OSTEOPOROSIS-CIM/GCST90274784.tsv.gz_leave_one_out.pdf]

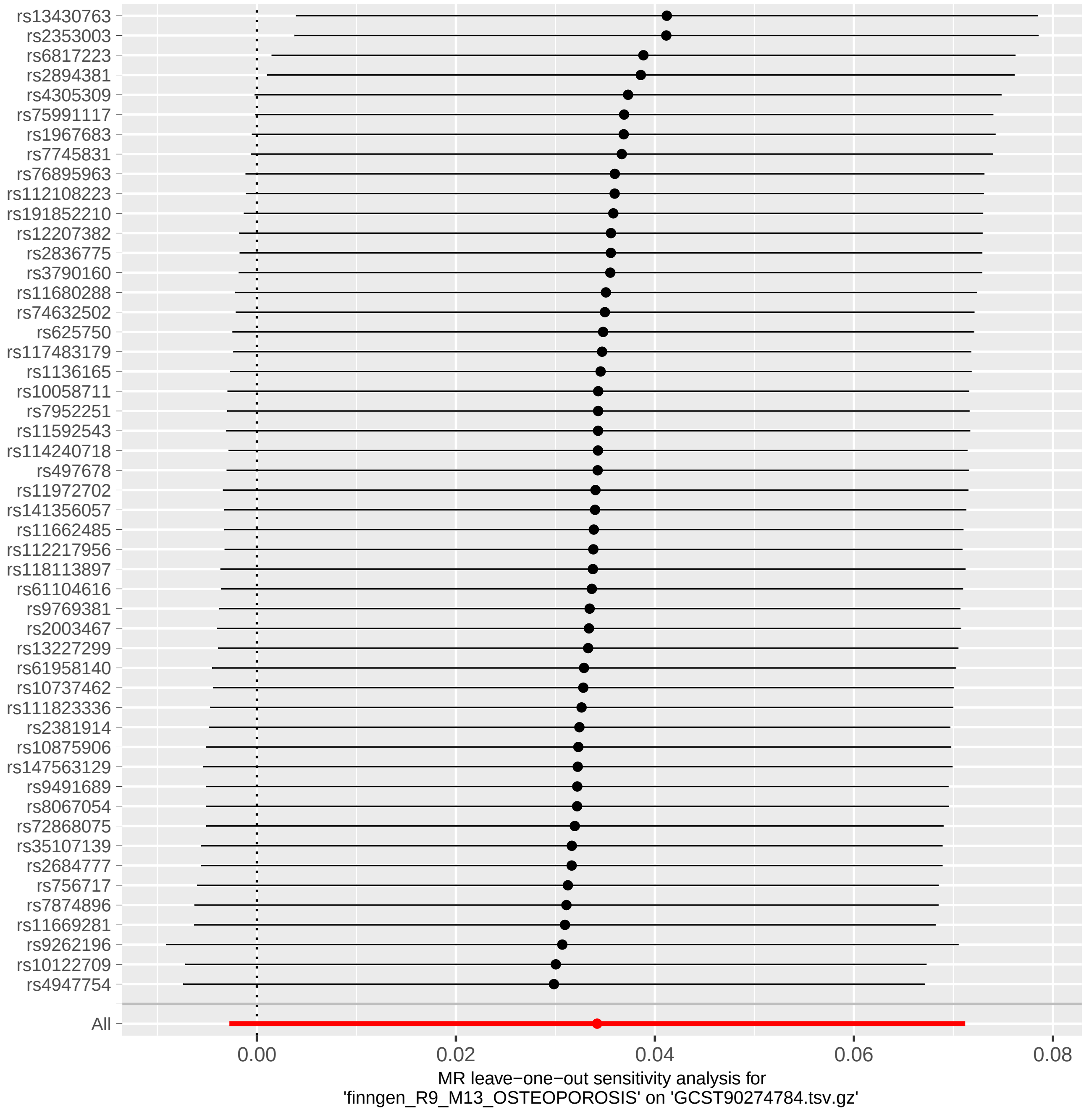

rs13430763
rs2353003
rs6817223
rs2894381
rs4305309
rs75991117
rs1967683
rs7745831
rs76895963
rs112108223
rs191852210
rs12207382
rs2836775
rs3790160
rs11680288
rs74632502
rs625750
rs117483179
rs1136165
rs10058711
rs7952251
rs11592543
rs114240718
rs497678
rs11972702
rs141356057
rs11662485
rs112217956
rs118113897
rs61104616
rs9769381
rs2003467
rs13227299
rs61958140
rs10737462
rs111823336
rs2381914
rs10875906
rs147563129
rs9491689
rs8067054
rs72868075
rs35107139
rs2684777
rs756717
rs7874896
rs11669281
rs9262196
rs10122709
rs4947754
All
0.00
0.02
0.04
0.06
0.08
MR leave−one−out sensitivity analysis for
'finngen_R9_M13_OSTEOPOROSIS' on 'GCST90274784.tsv.gz'
